# Risk of Venous Thromboembolism in Glioblastoma Patients

**DOI:** 10.7759/cureus.2678

**Published:** 2018-05-23

**Authors:** Gerald Lim, Clement Ho, Gloria Roldan Urgoti, Derek Leugner, Jay Easaw

**Affiliations:** 1 Department of Oncology, Tom Baker Cancer Centre, Calgary, CAN; 2 Radiation Oncology, BC Cancer Agency, Fraser Valley Centre, Surrey, USA; 3 EMS Data Science, AHS; 4 Medical Oncology, Cross Cancer, Edmonton, CAN

**Keywords:** radiotherapy, dvt, gbm

## Abstract

Background

Patients with cancer are at increased risk of venous thromboembolic events (VTE) with a particularly high prevalence in patients with glioblastoma (GB). We designed this current study to determine the incidence of symptomatic VTE in patients with GB undergoing first-line chemoradiotherapy and to develop a clinical score to help physicians identify those who are at the highest risk of VTE.

Methods

A retrospective study cohort included patients diagnosed with GBM treated with radical concurrent chemoradiotherapy between 2005 and 2010 in Southern Alberta. Descriptive statistics were used to characterize the patient population. A predictive value for VTE was assessed by comparing logistic models and using the area under the receiver operating characteristic curve.

Results

Twenty-three out of 115 patients (20%) experienced a symptomatic VTE. This complication was not associated with overall survival at two years (p=0.06, heart rate (HR)=1.61). Hypertension and smoking were associated with VTE (p-values 0.034 and 0.048, respectively). A scoring system with the following variables was developed to predict the likelihood of developing VTE: (1) Karnofsky performance status (KPS) - 70, 1 point; KPS < 70, 2 points; (2) Age – 45 to 60, 1 point; 61 to 70, 2 points; (3) Current smoking, 1 point; (4) Hypertension, 1 point. Patients with >3 points were 5 times more likely to develop a VTE.

Conclusions

In our population, our simple scoring system allows the identification of patients with GB receiving first-line therapy, who are at the highest risk of VTE. These results require validation in an independent series.

## Introduction

The association between cancer and venous thromboembolism (VTE) was described in the mid-nineteenth century by Trousseau [1-2]. Although the etiology is debated, suggested causes include the release of high levels of cytokines, acute phase proteins, and over-expression of tissue factors. Moreover, the activation of oncogenic pathways (RAS, EGFR, HER2, MET, SHH) and the loss of tumor suppressors (TP53, PTEN) alter the expression, activity, and release of coagulation factors [3]. Patients with malignancy have up to a 6.5-fold increased risk of developing VTE. The risk is particularly elevated in patients with brain cancers (HR 21.4) [4-5]. Among non-cancer causes of death, Khorana et al. identified VTE as the second-leading cause of death in cancer patients after infection [6].

VTE is of particular concern in patients with glioblastoma multiforme (GBM), where its incidence has been reported to be as high as 22% [5,7-8]. At present, there remains clinical equipoise regarding the relationship between VTE and overall survival. While one paper suggested no significant association between VTE and survival in patients with high-grade glioma [9], an epidemiological study of 9489 patients reported a 30% increased risk of death within two years of a VTE event [10]. Studies have been heterogeneous in their reporting of VTE incidences, with some identifying only symptomatic VTEs and others screening for asymptomatic cases. Most clinicians would consider symptomatic VTE to be clinically relevant, as it is not common practice to look for clots in asymptomatic individuals.

This is a population-based study assessing the incidence of symptomatic VTE in patients with GBM undergoing chemoradiotherapy and the clinical factors that could predict the likelihood of a patient developing VTE. Using these data, we developed a clinical score to help physicians identify GBM patients at the highest risk of developing clots.

## Materials and methods

Patient characteristics and treatments

This study was approved by the Calgary Health Region Ethics Board. We conducted a population-based evaluation of patients diagnosed with GB in Southern Alberta, Canada. Patients were identified by the provincial cancer registry and their clinical charts were reviewed. We identified 115 patients diagnosed with GBM (World Health Organization (WHO) grade IV glioma) between January 2005 and December 2010 who were offered radical concurrent chemoradiotherapy using temozolomide (75 mg/m2/day x six weeks) and radiotherapy prescribed to 60 Gy in 30 divided daily fractions during the same time period. Clinical characteristics analyzed included a past history of hypertension, dyslipidemia, diabetes mellitus, performance status (KPS score) at first consultation, and the presence of paresis at diagnosis. The presence of paresis was stratified as no paresis, paresis present but allowing independent mobilization, and severe paresis impairing independent mobilization. However, since a large majority of patients had no paresis, this variable was evaluated as paresis present or absent.

VTE detection and treatments

Symptomatic venous thromboembolic events, including either deep vein thrombosis and pulmonary embolism (PE) were identified using Doppler ultrasound or thin-cut computed tomography (CT) scan in accordance with institutional standards. Anticoagulation use for the treatment of symptomatic thromboembolism was confirmed using linked hospital discharge data and the provincial pharmacy network.

Data analysis

Clinical characteristics between groups of patients were compared using a two-sample t-test for continuous variables, and Pearson’s chi-square or Fisher's exact test for categorical variables, depending on sample size. Predictive value for VTE was assessed by comparing logistic models (with bootstrap) and further evaluated, using the area under the receiver operating characteristic (ROC) curve (AUC). Relative risks and odds ratios of developing VTE were estimated with 95% confidence intervals. All tests of significance were two-sided and p-values less than or equal to 0.05 were considered to be statistically significant.

Overall survival (OS) was calculated from the original date of consultation to date of death or last follow-up. Time to progression (TTP) was calculated from the date of first cancer center consultation to the date of disease progression or the last date of follow-up. Survival curves and corresponding standard errors were estimated using the Kaplan-Meier method. The association of clinical characteristics with OS was evaluated using the log-rank test. Prognostic value was analyzed using the Cox proportional hazard model (with bootstrap) for OS and TTP.

## Results

One hundred and fifteen patients with GBM received concurrent, postoperative, chemoradiotherapy between 2005 and 2010 in Southern Alberta, Canada. All had a consultation at the Tom Baker Cancer Center in Calgary. The median age was 57 years (range 23 to 83 years); 65% were male; 71% of patients underwent gross total (14%) or subtotal resection (57%); and all patients were treated with radical concurrent chemoradiotherapy (Table 1). Twenty percent of all patients experienced a symptomatic thromboembolic event (23 patients). There was no association between age and the development of VTE when age was dichotomized at the median (< 57 vs >57 years) (p=0.09). KPS score did not correlate with survival (p=0.74), likely due to the fact that patients who undertook radical concurrent chemoradiation were pre-selected for a minimum KPS of 70. A history of hypertension or smoking at diagnoses were associated with a higher likelihood of VTE with p-values of 0.034 and 0.048, respectively. The extent of surgery, dyslipidemia, and diabetes mellitus were not associated with the development of VTE or survival. Nine patients (39% of patients with VTE) had paresis; it impaired mobility in four cases. Due to the low number, the presence of paresis was analyzed as present (in any degree) vs no paresis and it was not associated with VTE or survival (Table 1).

**Table 1:** Patient characteristics, survival, and hazard ratios VTE: venous thromboembolism

Characteristics			All Patients	Patients with VTE		Patients without VTE		Association to VTE	Relative Risk for VTE			Median Survival in Months		Difference in Survival Experience (5yr)		Hazard Ratio	
			n(%)	n(%)		n(%)		p-value*	(95% CI)			median (95% CI)		p-value*		(95% CI)**	
Total			115 (100%)	23 (100%)		92 (100%)											
Age at Presentation																	
		Median (range)	57 (23-83)	61 (47-70)		56.5 (23-83)											
		<57	53 (46%)	7 (30%)		46 (50%)		0.092	0.51 (0.23, 1.15)			15.6 (12.2, 20)		0.1222		0.741 (0.51, 1.09)	
		>=57	62 (54%)	16 (70%)		46 (50%)			1.95 (0.87, 4.39)			14.2 (10.4, 17.4)				1.35 (0.92, 1.98)	
Gender																	
		Female	40 (35%)	8 (35%)		32 (35%)		0.591	1 (0.46, 2.15)			14.6 (11.6, 19.7)		0.4972		1.147 (0.77, 1.71)	
		Male	75 (65%)	15 (65%)		60 (65%)			1 (0.46, 2.15)			14.3 (11.5, 18.6)				0.872 (0.59, 1.3)	
Surgical Resection																	
		Biopsy/other	34 (30%)	10 (43%)		24 (26%)		0.144	Baseline			14.3 (11.2, 18.8)		0.8984		Baseline	
		Subtotal	65 (57%)	12 (52%)		53 (58%)			0.63 (0.3, 1.3)			14.2 (11.2, 19.4)				1.096 (0.71, 1.69)	
		Gross-total	16 (14%)	1 (4%)		15 (16%)			0.21 (0.03, 1.52)			16.7 (9.2, 20.6)				1.005 (0.54, 1.86)	
MGMT Promoter																	
		Unmethylated	56 (49%)	12 (52%)		44 (48%)		0.709	1.15 (0.55, 2.39)			12.5 (11.2, 14.8)		0.0050		1.728 (1.17, 2.54)	
		Methylated	59 (51%)	11 (48%)		48 (52%)			0.87 (0.42, 1.81)			19.2 (14.1, 22.6)				0.579 (0.39, 0.85)	
VTE																	
		No	92 (80%)	0 (0%)		92 (100%)		NA	NA			15.2 (12.6, 19.8)		0.3646		0.8 (0.49, 1.3)	
		Yes	23 (20%)	23 (100%)		0 (0%)						11.6 (7.7, 16)				1.249 (0.77, 2.02)	
Anticoagulation Regime																	
		None	92 (80%)	0 (0%)		92 (100%)		NA	NA			15.2 (12.6, 19.8)		0.3067		Baseline	
		LMWH	16 (14%)	16 (70%)		0 (0%)						9.7 (5.3, 14.1)				1.505 (0.87, 2.61)	
		Wafarin	7 (6%)	7 (30%)		0 (0%)						16.5 (7.7, 19.2)				0.874 (0.38, 2.01)	
KPS																	
		<=70	33 (29%)	7 (30%)		26 (28%)		0.837	1.09 (0.49, 2.4)			13.4 (9.4, 19.4)		0.7449		1.072 (0.71, 1.63)	
		>70	82 (71%)	16 (70%)		66 (72%)			0.92 (0.42, 2.03)			14.6 (12.2, 18.8)				0.933 (0.61, 1.42)	
Smoking																	
		No	92 (80%)	15 (65%)		77 (84%)		0.048	0.47 (0.23, 0.97)			14.8 (12.2, 18.8)		0.4810		0.844 (0.53, 1.35)	
		Yes	23 (20%)	8 (35%)		15 (16%)			2.13 (1.03, 4.41)			14.1 (9.6, 19.4)				1.184 (0.74, 1.9)	
Hypertension																	
		No	85 (74%)	13 (57%)		72 (78%)		0.034	0.46 (0.23, 0.93)			14.3 (12, 17.6)		0.1813		1.351 (0.87, 2.1)	
		Yes	30 (26%)	10 (43%)		20 (22%)			2.18 (1.07, 4.44)			15.5 (9.7, 26)				0.74 (0.48, 1.15)	
Dyslipidemia																	
		No	99 (86%)	20 (87%)		79 (86%)		0.893	1.08 (0.36, 3.21)			14.2 (12, 17.4)		0.5335		1.191 (0.69, 2.07)	
		Yes	16 (14%)	3 (13%)		13 (14%)			0.93 (0.31, 2.77)			16 (3.2, 25.1)				0.84 (0.48, 1.46)	
Diabetes Mellitus																	
		No	109 (95%)	21 (91%)		88 (96%)		0.345	0.58 (0.17, 1.91)			14.3 (12, 17.6)		0.0798		2.266 (0.89, 5.78)	
		Yes	6 (5%)	2 (9%)		4 (4%)			1.73 (0.52, 5.72)			14.8 (2.8, 0)				0.441 (0.17, 1.13)	
Paresis																	
		No	81 (70%)	14 (61%)		67 (73%)		0.261	0.65 (0.31, 1.36)			14.8 (12.6, 18.8)		0.4485		0.852 (0.56, 1.29)	
		Yes	34 (30%)	9 (39%)		25 (27%)			1.53 (0.73, 3.2)			11.9 (7.7, 20.2)				1.173 (0.78, 1.78)	

Eight of the 115 patients included in this series were enrolled in a Phase III, double-blind, clinical trial adding placebo/bevacizumab to the first-line treatment. Four patients developed VTE. However, it was not known whether patients were receiving the active drug or the placebo. Among the 23 patients that developed VTE, two presented after the initial consultation but before starting concurrent treatment, five were receiving chemoradiotherapy and 16 patients developed VTE after the conclusion of chemoradiotherapy.

It was not possible to discern from the chart review if VTE complications contributed to death in these patients. In 11 patients (48%), the time of VTE did not correlate with the documented time of progression or death (e.g. the two events were > four weeks apart). In six cases, the VTE diagnosis coincided with the time of recurrence/progression but occurred more than four weeks before death. In the remaining six cases, death (with or without the documented progression of disease) occurred within four weeks from the thromboembolic complication. Although VTE was not documented as the cause of death, it is possible that VTE contributed to death. Anecdotally, from the treating physicians, it was felt that all patients succumbed to their disease rather than an acute thromboembolic event.

**Figure 1:**
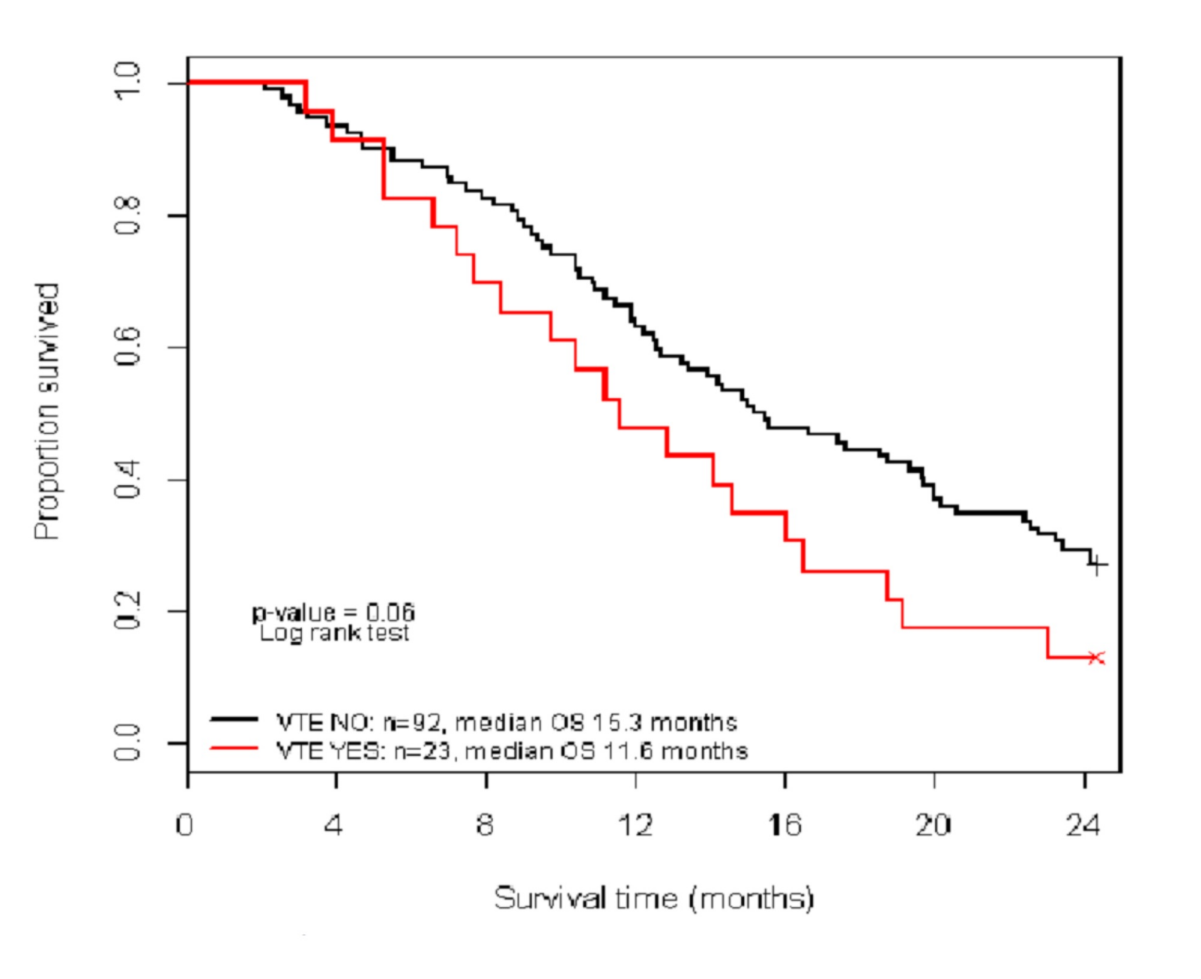
Kaplan Meier plot of two-year overall survival between VTE and non-VTE patients VTE: venous thromboembolism

The median OS for patients with and without VTE was 11.6 and 15.2 months, respectively. OS at two was not associated with a diagnosis of VTE (p= 0.06) (Figure 1).

Among the 23 patients who experienced symptomatic thromboembolic events, 16 were treated with low molecular weight heparin (LMWH) and seven were treated with warfarin. The median OS for patients treated with warfarin as compared with LMWH was 16.5 vs 9.7 months with overlapping ranges and no statistically significant difference.

**Figure 2:**
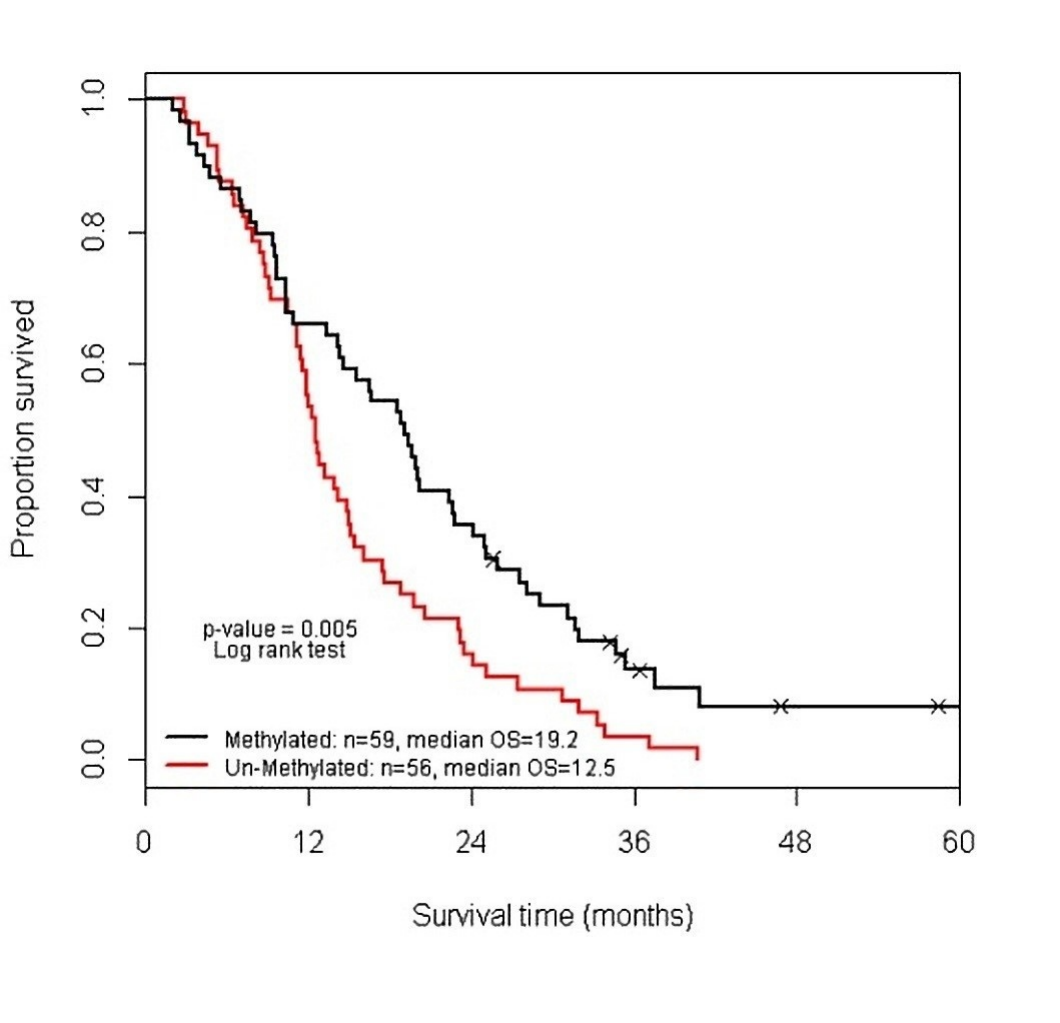
Overall survival of glioblastoma patients by methylation status

A molecular tumor analysis showed almost equal distribution between methylated- and unmethylated-O-6-methylguanine-DNA methyltransferase (MGMT) promoter status (51% and 49%, respectively). This parameter was not associated with VTE occurrence but OS at five years was significantly better in patients with a methylated-MGMT promoter (HR 0.579, p=0.005), compared with patients whose tumors had unmethylated MGMT promoter (Figure 2).

Predicting the likelihood of developing a symptomatic clot

Variable inclusion and nested model comparisons were evaluated using the Wald and log-likelihood statistics, model prediction performance was evaluated using the area under the ROC curve (AUC). For choosing the best model, statistical penalization methods and variable selection models (Enet, NEnet, Lasso; utilizing cross-validation) were compared to the stepwise and best subsets of a logistic regression model; AUC is reported for interpretability.

A practical score that is simple to perform in routine clinical situations was developed. The score was based on four variables:

(1) Karnofsky performance status: 70 = 1 point; < 70 = 2 points; otherwise, 0 points

(2) Age: 45 to 60 years = 1 point; 61 to 70 years = 2 points; otherwise, 0 points

(3) Smoking at diagnosis: Yes = 1 point, No = 0 points

(4) Hypertension history: Yes = 1 point, No = 0 points

These scores are summed for a total score per patient (range, 0 to 6) with AUC=0.758 (95% CI: 0.668, 0.832) and p-value=0.001. For every point increase, the odds of a patient being diagnosed with a symptomatic VTE increased by 2.2 (95% CI: 1.3, 3.7; p=0.002). A validated cut point allowing the categorization of patients into low- and high-risk groups was ≤2 vs. >2 points. These two risk categories have AUC = 0.739 (95% CI: 0.649, 0.817) and p <0.001. Patients with scores >2 points (high risk of a clot) had a five-times (95% CI: 2.3, 11.1) higher risk of a symptomatic VTE compared to patients with scores ≤2.

## Discussion

Glioblastoma multiforme is the most common primary brain tumor in adults. Despite surgery, followed by radiation therapy and chemotherapy, most patients with GBM go through a rapid and disappointing clinical course with a median survival of just over a year [11-12]. Unfortunately, the poor clinical course for these patients is worsened by a high rate of symptomatic VTEs and other complications. In this retrospective, population-based review, 20% of patients developed a symptomatic VTE. It could be useful to identify beforehand the subset of patients at highest risk of VTE. It is unclear if the development of a VTE signifies a worse prognosis for OS, but the simple predictive algorithm proposed identified patients with a five-fold higher risk of VTE. Although the median survival of patients in this study with and without VTE was 11.6 months and 15.3 months, respectively, this was not statistically significant (P=0.06). The small sample size may have prevented this difference from reaching statistical significance. Whether prophylactic anticoagulation therapy would improve the quality of life or OS needs further validation.

Despite the very high incidence of VTE/PE, it would be both impractical and potentially unsafe to prophylactically anticoagulate every GBM patient [5,13]. The early terminated PRODIGE study showed that the primary prophylaxis of GB patients caused a trend towards diminishing VTE events at the cost of an increased incidence of intracranial hemorrhage and without a survival benefit [13]. However, our data suggest that it may be possible to identify GBM patients at the highest risk for thromboembolic complications, thereby allowing the targeted use of anticoagulants in a carefully selected group of GBM patients.

In this study, our goal was to develop a simple practical scoring system specific to GBM patients that could identify those patients at the highest risk of developing VTE. Among all the variables examined, only smoking and hypertension independently predicted a higher risk of clot formation. However, when combined with age and KPS, these four variables produced a highly predictive score for symptomatic clot formation in our patient population. Our data suggest that a clot score greater than 2 points suggests that the patient has a five-fold increased risk of developing a symptomatic VTE. Higher risk patients, including patients with GBM, are routinely counseled about the signs and symptoms of VTEs and to avoid long periods of stasis and other activities that predispose clots. Prophylactic anticoagulation in all GBM patients is controversial but perhaps targeting a selected subset of GBM patients with a high clotting score on our scale might be beneficial. The merits of this intervention in this subset of patients warrants further research.

Our scoring system identified in this report includes just four variables: KPS, age, smoking, and hypertension. The association between known cardiovascular risk factors (i.e. metabolic syndrome, obesity, hypertension, diabetes) and VTE has been previously recognized [14]. Mechanistically, smoking and hypertension could result in endothelial injury and loss of elasticity, thus resulting in an increased risk of clot formation. Patients with poorer performance status would likely be less mobile and less independent in their functioning. The lack of mobility would result in stasis, a well-known risk factor for developing clots. Older age could contribute in such a manner in addition to having a vascular system that was less healthy and more prone to injury and subsequent clots.

This scoring system is a first attempt to create a simple practical scoring system to identify GBM patients at the highest risk of developing a blood clot. However, we appreciate that this study has a number of limitations, including its retrospective design with a low number of patients that precludes further subgroup analysis and may account for the lack of a statistical significance of some results. Also, the described population is a selected group of patients, already with good KPS, enough to be candidates for concurrent chemoradiotherapy treatment and these results cannot be extrapolated to the total population of patients with GBM. We did not collect information about the concurrent use of drugs that could alter DVT risk (i.e. ASA, clopidogrel, hormonal replacement therapy). Although there was no statistical difference in outcomes based on DVT treatment (low molecular weight heparin (LMWH) vs warfarin), the small number of patients precluded a meaningful analysis in this area. This certainly would be of particular interest and has not been well studied. Despite the high incidence of VTE in this population, patients with GBM are usually absent or underrepresented in studies evaluating anticoagulation. The recently published randomized control study of tinzaparin versus warfarin in patients with cancer does not address the issue in this population [15].

Although a prospective trial to evaluate this score would be optimal, our next goal is to validate this score using a larger dataset, which will include GBM patient cohorts from other Canadian centers. If validated, this score could then be evaluated in a prospective clinical study.

## Conclusions

GBM is the most common malignant primary brain tumor in adults. Patients with GBM have a poor prognosis and are at a high risk of developing symptomatic VTE. In this study, 20% of patients were found to have a symptomatic VTE. It is unclear if the diagnosis of the disease predicts poorer OS. However, a simple scoring system based on age, KPS, smoking, and hypertension histories, allowed the identification of patients with GBM receiving first-line therapy, who were at the highest risk of VTE. These results require validation in an independent series.
